# Impact of prior aspirin use on left ventricular function in ST-elevation myocardial infarction patients undergoing primary percutaneous coronary intervention: An echocardiographic evaluation

**DOI:** 10.34172/jcvtr.33184

**Published:** 2024-09-20

**Authors:** Yosef Yosefzadeh, Mahdokht Rezaei, Abbas Allami, Ali Hosseinsabet

**Affiliations:** ^1^Department of Cardiology, Bouali Hospital, Qazvin University of Medical Sciences, Qazvin, Iran; ^2^Department of Cardiology, Faculty of Medicine, Bouali Hospital, Qazvin University of Medical Sciences, Qazvin, Iran; ^3^Department of Infectious Diseases, Faculty of Medicine, Bouali Hospital, Qazvin University of Medical Sciences, Qazvin, Iran; ^4^Department of Cardiology, School of Medicine, Tehran Heart Center, Tehran University of Medical Sciences, Tehran, Iran

**Keywords:** Myocardial infarction, Echocardiography, Percutaneous coronary intervention, Aspirin, Heart ventricles

## Abstract

**Introduction:**

Previous studies have investigated the potential influence of prior aspirin use on cardiac function in patients with ST-elevation myocardial infarction (STEMI) who undergo primary percutaneous coronary intervention (PPCI). However, the results from these studies have been conflicting. This study aimed to investigate whether prior aspirin use affects left ventricular (LV) function in these patients using echocardiography.

**Methods:**

The study included 260 consecutive STEMI patients, who were divided into two groups based on the presence or absence of prior aspirin use. Echocardiographic parameters, such as maximal left atrial (LA) size, LV ejection fraction (LVEF), early diastolic velocity (e’), E/A ratio, and E/e’ ratio, were assessed within 72 hours of admission.

**Results:**

Aspirin users had an older age compared to non-users, as well as lower body mass index and renal function. They also had a greater history of hypertension and were more likely to be taking statins, angiotensin-converting enzyme inhibitors/angiotensin receptor blockers, and calcium channel blockers. There were no significant differences in LVEF, maximal LA size, E/A ratio, E/e’ ratio, and deceleration time between aspirin users and non-users. e’ wave was marginally lower in aspirin users (*P*=0.054). After controlling for confounding variables, the previous use of aspirin did not show a significant impact.

**Conclusion:**

Prior aspirin use in STEMI patients does not have a significant impact on LV echocardiographic parameters. Our conclusions remained consistent even after adjusting for potential confounders.

## Introduction

 Acute myocardial infarction (AMI) remains a significant cause of morbidity and mortality worldwide, despite advances in medical management. Primary percutaneous coronary intervention (PPCI) is a crucial treatment strategy for timely restoration of blood flow to the coronary arteries. Aspirin is a cornerstone of antiplatelet therapy for secondary prevention of cardiovascular disease (CAD)^1-4^, but its role in primary prevention during the PPCI era remains uncertain.^5-7^ Prior studies have explored the potential influence of prior aspirin use on cardiac function in STEMI patients undergoing PPCI, with conflicting results. Some have suggested protective effects^8^, while others have reported no significant impact^9^ or even harmful effects.^10-12^ For example, Rich et al found that prior aspirin use was not linked to increased mortality but may increase the risk of recurrent myocardial infarction.^12^ Santopinto et al found that prior aspirin use may be an independent risk factor for poor prognosis.^10^ Gum et al investigated the association between aspirin use and left ventricular (LV) function. The study showed that aspirin users had a higher prevalence of reduced LV ejection fraction (< 40%) compared to non-users, as well as increased rates of stress-induced ischemia on echocardiography.^13^ The studies suggest that aspirin use is linked to both reduced heart attack risk and higher mortality after a heart attack.

 The relationship between prior aspirin use and outcomes after AMI is complex and warrants further investigation. This study aimed to investigate the prevalence and severity of post-PPCI LV echocardiographic parameter abnormality among STEMI patients and evaluate the influence of prior aspirin use on LV function in this population. By utilizing comprehensive echocardiographic assessment, the study seeks to provide a detailed understanding of the potential impact of prior aspirin use on cardiac mechanics in the setting of AMI.

## Materials and Methods

###  Patient selection

 We conducted a prospective observational study of STEMI patients undergoing PPCI from June 2017 to March 2021 in two tertiary referral cardiovascular centers with PPCI services. Consecutive patients admitted with STEMI were enrolled in the study. A total of 260 patients with STEMI were included in the study. Out of these, 45 patients had prior aspirin use, while 215 patients had no prior aspirin use. These patients were recruited consecutively from among those who underwent PPCI. The information of 155 patients has already been published in research with other objectives.^14,15^

 STEMI was defined according to the third universal definition of myocardial infarction (MI).^16^ PPCI was performed within 12 hours of symptom onset, and for patients with symptoms lasting between 12 to 24 hours if pain persisted at admission. AMI patients were treated following the latest recommendations.^2^ Detailed clinical data, including demographics, medical history, laboratory findings, and echocardiographic parameters, were collected. Medical history variables such as diabetes mellitus, hypertension, and hyperlipoproteinemia were assessed due to their significant role in the development of CAD. A family history of early CAD was defined as having a first-degree relative with CAD onset at or before the age of 50 for males and 60 for females.^17^ Exclusion criteria for both patient groups were: chest pain continuously for ≥ 24 hours duration, cardiac rhythms other than sinus rhythm, a history of MI or CAD, congenital heart diseases, pericardial disease, cardiomyopathies, poor echocardiography windows, autoimmune disorders, liver diseases, thyroid disorders, cancer, estimated glomerular filtration rates (GFR) < 45 mL/min/1.73m^2^ by the MDRD GFR Equation, and more-than-moderate valvular regurgitation. Venous blood samples were collected from the patients upon their admission to the hospital. These blood samples were then evaluated to assess the patient’s creatinine and blood urea nitrogen levels. Patient records were assessed for prior aspirin use, which was defined as consumption within the past month. The two patient groups were then divided based on their history of aspirin usage. The primary outcome will involve evaluating LV function parameters, including LVEF, early diastolic mitral annulus velocity (e’), the early mitral inflow velocity to early diastolic mitral annulus velocity ratio (E/e’ ratio), and the early to atrial filling velocity ratio (E/A ratio) and maximal LA volume index. This research proposal was approved by the institutional review board, and all patients provided written informed consent upon their admission.

###  Echocardiographic measures 

 All echocardiographic assessments were done using Affiniti 50 and 70 Philips Ultrasound Machine (Philips Healthcare, Andover, MA, USA) using the S5- I probe. For STEMI patients. All transthoracic echocardiographic examinations were performed within 72 hours of admission. During the echocardiographic examination, the patients were positioned in the left lateral decubitus position. They were also monitored using a single-lead ECG to ensure that there were no arrhythmias that could affect the accuracy of the measurements.

 In the parasternal long-axis view, measurements were taken of the septal wall thickness, posterior wall thickness, left ventricular end-systolic diameter (LVESD), and left ventricular end-diastolic diameter (LVEDD). These measurements were used to calculate LV mass. Atrial and ventricular volumes were measured relative to body area (BSA) to ensure comparability between patients. The formula for calculating LV mass is: LV mass (g) = 0.8 × [1.04 × (LVEDD + Septum + Posterior)^3^ - LVEDD^3^] + 0.6. Here, LVEDD represents the end-diastolic LV inner diameter, Septum represents the interventricular septal thickness, and Posterior represents the posterior wall thickness.^18^

 In the apical four-chamber view, E and A mitral flow waves were evaluated using pulsed-wave Doppler, and their peaks were recorded. Additionally, a custom four-chamber apical view was used to maximize LA size and avoid LA foreshortening for LA volume measurement. E-wave deceleration time was also recorded. Then, the maximum velocities in the septal (e’) and lateral (a’) mitral annulus were measured by tissue Doppler. This yielded an average value of three cardiac cycles for each patient, with their means reported and utilized to calculate the E/averaged e’ ratio. Finally, LV end-systolic and end-diastolic volumes and LVEF were obtained. These measurements were obtained from the apical four-chamber and two-chamber views using the biplane-modified Simpson’s method.^19^ All echocardiographic measurements were conducted following the guidelines set forth by the American Society of Echocardiography.^20^

###  Statistical analysis 

 To determine the required sample size for the study, we used the following formula:

 n = (Z₁-α/₂)^2^ × P(1-P) / d^2^, Where: n = the required sample size, Z₁-α/₂ = the standard normal variate at the desired level of significance (1.96 for a 95% confidence level), P = the proportion of patients with prior aspirin use (0.20) and d = the desired margin of error (0.05 for a 5% margin of error). Plugging in the values, the required sample size for the study is approximately 246 patients. Of these, 49 patients (0.20 × 246) are expected to have prior aspirin use, and the remaining 197 patients are expected to have no prior aspirin use.

 The categorical data were compared using either the chi-square test or Fisher’s exact test, as appropriate. The continuous variables are presented as the mean ± standard deviation (SD). For the comparison of two groups of continuous data, the Student’s t-test was employed. To account for potential confounders such as age, body mass index (BMI), hypertension history, statin use, calcium channel blocker use, angiotensin-converting enzyme (ACE) inhibitors/angiotensin receptor blocker (ARB) use, two-vessel disease, and GFR multiple regression analysis was applied. Statistical analyses were performed using SPSS Statistics V 27 (IBM Corp., Armonk, NY). A p-value < 0.05 was considered statistically significant.

## Results

 The clinical, demographic, and laboratory data of the study population are summarized in Table 1. Patients who had previously used aspirin were significantly older, with a mean age of 63 years, compared to those who had not used aspirin, with a mean age of 57 years (*P* < 0.001). Additionally, patients with aspirin usage had a significantly lower mean BMI compared to those without aspirin use, which was statistically significant (*P* = 0.043). Hypertension was significantly more prevalent in the aspirin group (*P* < 0.001), as were the use of statins (*P* = 0.002), calcium channel blockers (*P* = 0.005), and ACEI/ARB (*P* = 0.007). GFR was significantly lower in the aspirin group (*P* = 0.004). Among aspirin non-users, 42 cases (19.5%) had two-vessel disease, while among aspirin users, 16 cases (35.6%) were affected. This disparity was statistically significant (*P* = 0.019). Overall, the results suggest that aspirin usage is associated with older age, lower weight, and a lower BMI. Additionally, aspirin users are more likely to have hypertension and are more frequently treated with certain cardiovascular medications.

**Table 1:** Patient characteristics

**Variable**		**History of aspirin use**		***P*** ** value**
		**No (n=215)**	**Yes (n=45)**	
Age (y)		57 ± 11	63 ± 10	< 0.001*
Male sex (%)		171 (79.5)	36 (80.0)	0.944
Height (kg)		170 ± 9	169 ± 8	0.525
Weight (m)		79 ± 14	75 ± 11	0.030*
Body mass index (kg/m^2^)		27.61 ± 4.13	26.27 ± 3.43	0.043*
Obesity (%)	Obese	54 (25.1)	5 (11.1)	0.096
	Overweight	102 (47.4)	23 (51.1)	
	Other^†^	59 (27.4)	17 (37.8)	
Body surface area (m^2^)		1.93 ± 0.21	1.87 ± 0.16	0.053
Systolic blood pressure (mm Hg)		126 ± 23	130 ± 21	0.260
Diastolic blood pressure (mm Hg)		79 ± 16	81 ± 13	0.481
Diabetes mellitus (%)		56 (26.0)	15 (33.3)	0.318
Hypertension (%)		66 (30.7)	28 (62.2)	< 0.001*
Hyperlipoproteinemia (%)		42 (19.5)	10 (22.2)	0.682
Family HX of CAD (%)		42 (19.5)	6 (13.3)	0.330
Cigarette smoking (%)		88 (40.9)	15 (33.3)	0.343
Involved coronary vessel (%)	LAD	165 (76.7)	31 (68.9)	0.161
	LCX	18 (8.4)	8 (17.8)	
	RCA	32 (14.9)	6 (13.3)	
LAD (%)		156 (72.6)	32 (71.1)	0.844
LCX (%)		71 (33.0)	18 (40.0)	0.370
RCA (%)		102 (47.4)	21 (46.7)	0.925
Single-vessel disease (%)		137 (63.7)	24 (53.3)	0.192
Two-vessel disease (%)		42 (19.5)	16 (35.6)	0.019*
Three-vessel disease (%)		36 (16.7)	5 (11.1)	0.346
Blood urea nitrogen (mg/dL)		15.30 ± 5.18	15.01 ± 3.71	0.734
Creatinine (mg/dL)		0.96 ± 0.21	1.03 ± 0.23	0.037
MDRD eGFR Equation (mL/min/1.73 m^2^)		82.75 ± 18.96	73.84 ± 18.77	0.004*
Hx of statin usage (%)		15 (7.0)	10 (22.2)	0.002*
Hx of beta-blocker usage (%)		16 (7.4)	7 (15.6)	0.081
Hx of calcium channel blocker usage (%)		7 (3.3)	6 (13.3)	0.005*
Hx of ACEI/ARB usage (%)		31 (14.4)	14 (31.1)	0.007*
Hx of nitrate usage (%)		1 (0.5)	0 (0)	0.647
Hx of oral antidiabetic usage (%)		37 (30.8)	9 (30.0)	0.929
Hx of insulin usage (%)		5 (4.2)	2 (6.7)	0.561

Continuous variables are expressed as the mean ± standard deviation. Categorical variables are presented as frequencies (%). ACEI: angiotensin-converting enzyme inhibitors, ARB: angiotensin receptor blocker, CAD: Coronary Artery Disease, eGFR: An estimated glomerular filtration rate, Hx: History, LAD: left anterior descending, LCX: left circumflex artery, MDRD: Modification of Diet in Renal Disease formula, RCA: right coronary artery * *P* < 0.05. ^†^: Other: Underweight and Normal weight

 The echocardiographic assessment revealed that there were no statistically significant differences in most parameters between patients with a history of aspirin use and those without. The LVEF was slightly higher in the aspirin group (42.28% ± 8.55) compared to the non-aspirin group (41.40% ± 8.14), but this difference was not statistically significant (*P* = 0.514). The details of standard echo findings and volume measurements for the participants in this research can be found in Table 2.

**Table 2:** Echocardiographic data

**Variable**		**History of aspirin use**		***P*** ** value**
		**No (n=215)**	**Yes (n=45)**	
Simpson’s ejection fraction (%)		41.40 ± 8.14	42.28 ± 8.55	0.514
LVEF (%)	normal & mildly abnormal (≥ 40)	147 (68.4)	32 (71.1)	0.718
	moderately & severely abnormal (< 40)	68 (31.6)	13 (28.9)	
LV mass index (g/m^2^)		88.81 ± 22.93	96.17 ± 24.86	0.055
LV end-diastolic diameter (cm)		52 ± 6	52 ± 8	0.650
LV end-systolic diameter (cm)		36 ± 6	36 ± 7	0.762
Septum (cm/s)		9.0 ± 1.2	9.2 ± 1.2	0.282
Posterior (cm/s)		8.7 ± 0.9	8.9 ± 1.2	0.189
Peak E Velocity (cm/s)		67.31 ± 20.12	66.67 ± 21.07	0.849
Peak A Velocity (cm/s)		67.40 ± 19.00	72.05 ± 21.44	0.154
e septum (cm/s)		6.51 ± 1.77	6.09 ± 1.64	0.140
a septum (cm/s)		8.04 ± 1.86	8.57 ± 2.09	0.090
e lateral (cm/s)		8.17 ± 2.44	7.41 ± 2.24	0.058
a lateral (cm/s)		9.52 ± 2.40	10.23 ± 2.56	0.079
Mean e (cm/s)		7.34 ± 1.87	6.75 ± 1.73	0.054
E/A ratio		1.07 ± 0.48	0.96 ± 0.37	0.151
EDV index (mL/m^2^)		47.80 ± 9.46	49.69 ± 14.59	0.414
ESV index (mL/m^2^)		27.20 ± 7.44	28.00 ± 10.11	0.650
MLA index (mL/m^2^)		25.65 ± 7.54	27.61 ± 10.31	0.231
MLA Index (mL/m^2^)	normal	189 (87.9)	35 (77.8)	0.074
	abnormal	26 (12.1)	10 (22.2)	
E/e’ ratio		9.61 ± 3.94	10.08 ± 3.40	0.461
E/e’ ratio	normal	131 (61.2)	24 (54.5)	0.247
	borderline	70 (32.7)	15 (34.1)	
	increased	13 (6.1)	5 (11.4)	
DT (ms)		194.23 ± 56.92	191.84 ± 59.14	0.802
DT (ms)	≤ 140	36 (17.6)	9 (20.5)	0.758
	140-160	23 (11.2)	7 (15.9)	
	160- 220	87 (42.4)	16 (36.4)	
	> 220	59 (28.8)	12 (27.3)	

DT: Deceleration time, E/A ratio: Early to atrial filling velocity ratio, EDV index: End-diastolic volume index, E/e’ ratio: Early mitral inflow velocity to early diastolic mitral annulus velocity ratio, ESV index: End-systolic volume index, LA: left atrium, LV: Left ventricular, LVEF: Left ventricular ejection fraction, MLA index: Maximal LA volume

 In this study, measurements of LV end-diastolic and end-systolic diameters, septal and posterior wall thicknesses, peak E and A velocities, the early to atrial filling velocity ratio (E/A ratio), and the early mitral inflow velocity to early diastolic mitral annulus velocity ratio (E/e’ ratio) were identical between patients with and without a history of aspirin use. The mean early diastolic velocity (e’) was marginally lower in the aspirin group (6.75 cm/s ± 1.73) compared to the non-aspirin group (7.34 cm/s ± 1.87), approaching statistical significance (P = 0.054). Volumes such as end-diastolic volume (EDV) index, end-systolic volume (ESV) index, and their respective indices, as well as maximal LA volume, showed no significant differences between the two groups. In summary, echocardiographic parameters evaluated in this study did not show significant differences between patients with and without a history of aspirin usage, indicating that aspirin does not have a marked impact on these specific cardiac structural and functional measurements. After controlling for confounding variables (such as age and prior statin use), the previous use of aspirin did not show a significant impact on the parameters of LV function, suggesting that aspirin does not have a significant impact on these cardiac measurements even after adjusting for potential confounders.

## Discussion

 This study aimed to assess the impact of previous aspirin usage on LV function in patients with STEMI undergoing PPCI. In our analysis, we found no significant differences between the prior aspirin use group and non-aspirin use group regarding LVEF, a marker of LV systolic function; mean early diastolic mitral annulus velocity (e’), an indicator of diastolic LV function; and the E/e’ ratio, which reflects LV filling pressure. These findings suggest that prior aspirin use did not significantly affect LV function in this patient population. Our conclusions remained consistent even after adjusting for potential confounders. This study is, to our knowledge, the second echocardiographic evaluation of LV function in this particular context, following the initial assessment conducted by Gum et al.^13^

 The lack of significant impact of prior aspirin use on LV function observed in this study may be attributed to several factors. Firstly, it supported the idea that aspirin did not lead to a worsening of LV function. Secondly, the protective effects of aspirin in the setting of STEMI may be primarily related to its antiplatelet and antithrombotic properties, which are more directly associated with the prevention of acute coronary events, rather than having a direct influence on cardiac structure and function. The timely restoration of blood flow through PPCI may have been the predominant factor in determining the recovery of LV function, regardless of prior aspirin use.^21^ Additionally, the study population may have had varying degrees of underlying cardiovascular disease and risk factors, which could have influenced the cardiac function independent of aspirin use. Factors such as the duration and adherence to aspirin therapy, as well as the presence of other comorbidities, were not accounted for in the current analysis, which may have contributed to the lack of a significant association.

 Two previous studies have investigated angiographic differences between aspirin users and non-users in this context. Niccoli et al’s (2010) research found that chronic aspirin therapy was associated with a decrease in angiographic thrombus grade in first-time STEMI patients treated with PPCI, suggesting the potential benefits of aspirin use in reducing thrombotic burden.^22^ Geraiely et al (2018) investigated the impact of prior aspirin intake on STEMI patients undergoing PPCI and reported worse outcomes for aspirin users, including higher rates of totally occluded infarct-related artery and lower postprocedural TIMI flow grade 3 (poorer angiographic features). Aspirin consumption was associated with increased long-term mortality and major adverse cardiac events in these patients. The findings imply that STEMI development despite chronic aspirin intake may be due to more vulnerable coronary plaques, leading to unfavorable outcomes.^23^ Geraiely et al reported a higher incidence of 3-vessel disease in the aspirin users compared to the aspirin non-users and a greater likelihood for patients in the aspirin users to have a history of previous PCI. However, in Niccoli et al study, patients with a history of CAD were excluded, which may have influenced the differences in results between the two studies. Our study observed that the aspirin users had notably older patients than the aspirin non-users, which aligns with Geraiely et al’s findings. In our study, aspirin usage was linked to older age, lower weight, and a lower BMI. Furthermore, aspirin users were more prone to having hypertension and two-vessel disease, and they were more commonly prescribed specific cardiovascular medications. These observations align closely with the results reported by Gum et al.^13^

 This study aimed to explore whether the seemingly conflicting findings between previous studies on STEMI patients undergoing PPCI could be explained by differences in echocardiographic parameters post-PPCI. The results indicate that the contrasting outcomes observed between aspirin users and non-users cannot be predicted through echocardiography performed after PPCI. This study underscores the complex, multifactorial nature of the relationship between aspirin use, heart attack risk, and post-heart attack outcomes. Further research is required to unravel the underlying mechanisms and identify patient-specific factors that may influence the effects of aspirin on cardiovascular disease.

 It should be noted that the echocardiographic assessment occurred at a single time point during the acute phase of STEMI. A more comprehensive understanding of how prior aspirin use may impact cardiac function recovery over time might require longitudinal evaluation. Furthermore, the utilization of more contemporary methods to measure changes in LV function may yield a different result, potentially revealing a distinction between the two groups. Our study population included patients with 2VD/3VD, which may have influenced echocardiographic data and affected our findings. We did not find significant correlations between the number of diseased vessels (2VD/3VD vs. single-vessel disease) and changes in LV function. Future studies could use advanced imaging modalities like cardiac MRI or CTA to non-invasively assess coronary artery disease extent, or employ statistical techniques that account for potential confounding factors related to residual stenotic coronary arteries. Additionally, patients with multivessel disease and prior aspirin use may have experienced silent myocardial infarctions in the past, which could confound results.

## Conclusion

 In conclusion, this study found no significant influence of previous aspirin intake on LV function in STEMI patients undergoing PPCI. Further research with larger sample sizes, longer follow-ups, and more extensive evaluations of patient characteristics and comorbidities may be necessary to further explore the potential role of aspirin in preserving or enhancing cardiac function in the context of STEMI.

## Acknowledgements

 Not applicable.

## Competing Interests

 The authors declare no competing interests.

## Ethical Approval

 The study involving human participants followed the ethical guidelines and standards set by the institutional and/or national research committee, in accordance with the principles outlined in the 1964 Helsinki Declaration and any subsequent amendments, or adhered to comparable ethical standards. The study protocol was reviewed and approved by the Ethics Committee affiliated with Qazvin University of Medical Sciences (IR.QUMS.REC.1400.127). Written informed consent for publication of their clinical details was obtained from the patients.
